# Recent Applications and Future Perspectives of Chemiluminescent and Bioluminescent Imaging Technologies

**DOI:** 10.1021/cbmi.2c00002

**Published:** 2023-04-13

**Authors:** Islam Mohamed Mostafa, Abubakar Abdussalam, Yuriy Tymofiiovych Zholudov, Dmytro Viktorovych Snizhko, Wei Zhang, Morteza Hosseini, Yiran Guan, Guobao Xu

**Affiliations:** † State Key Laboratory of Electroanalytical Chemistry, Changchun Institute of Applied Chemistry, Chinese Academy of Sciences, Changchun, Jilin 130022, P.R. China; ‡ University of Science and Technology of China, Hefei 230000, P.R. China; § Analytical Chemistry Department, Faculty of Pharmacy, Minia University, Minia 61519, Egypt; ∥ Department of Chemistry, College of Natural and Pharmaceutical Sciences, Bayero University, PMB 3011, Kano 700006, Nigeria; ⊥ Laboratory of Analytical Optochemotronics, Biomedical Engineering Department, Kharkiv National University of Radio Electronics, Kharkiv 61166, Ukraine; # Nanobiosensors Lab, Department of Life Science Engineering, Faculty of New Sciences & Technologies, University of Tehran, Tehran 1439817435, Iran

**Keywords:** Chemiluminescence, Bioluminescence, *In vivo*, Imaging, Biomolecules, Noninvasive, Luciferase, Biochemical analysis

## Abstract

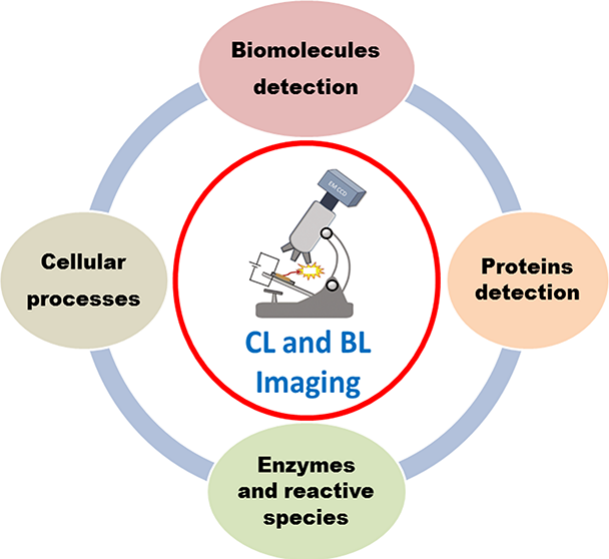

Imaging technologies based on chemiluminescence (CL) and bioluminescence (BL) have seen a tremendous growth in the past decade due to their extensive contributions to biochemical analysis and biomedical science. CL and BL imaging technologies have many advantages over commonly used imaging techniques (such as fluorescence and electrochemical systems). CL and BL proceed without an external light source, thus avoiding photobleaching, high interference of background signal, and autofluorescence. Furthermore, CL and BL analytical techniques are characterized by their low detection limit, high selectivity, short assay time, and simple instrumentation. Recently, CL-based imaging technology has been applied successfully for the determination and *in vivo* imaging of several significant analytes. Meanwhile, an innovative BL imaging technology has been established for the noninvasive real-time tracking of different biomolecules relying on the interaction between luciferase and its substrate. In the current review, we discuss the recent applications of CL and BL imaging approaches over the past five years. Finally, we also discuss the current state of progress and the prospects for CL and BL imaging systems.

## 1 Introduction

Imaging technology has contributed significantly to the imaging and detection of bioinformatics in living subjects.^1^ With great progress in optical-based imaging technologies, recent decades have witnessed important progress in their utilization in biomedical implementations and discovering the molecular basis of life.^2^ Moreover, imaging approaches offer a new cytological tool for decrypting living cell secrets and revealing previously undiscovered metabolic pathways, including disease-formation pathways. Recently, imaging approaches have been established for noninvasive imaging of different significant analytes inside the cell.^3−9^ Fluorescence^5,6^ and electrochemical^9,10^ imaging approaches are the most commonly applied approaches for imaging purposes. Nevertheless, the autofluorescence background and the need for high-power laser irradiation are the main drawbacks of fluorescence-based imaging approaches that can induce significant background response (reduce imaging contrast) and cause permanent damage to biological materials.^11−13^ Moreover, electrochemical-based imaging methods are complicated and difficult to be applied in biological research.^14^ Also, there are many successful applications involving scanning single cells with a microelectrode in scanning electrochemical microscopy (SECM) mode; however, the main drawback of SECM is its poor temporal resolution attributed to the necessity to scan the sample and topography of the cell rebuilding from electrochemical activity mapping, despite the fact that soft microelectrode arrays represent a promising approach.^15,16^ As a result, the exploration for a highly sensitive, simple, selective, and noninvasive methods for imaging biological analytes is still ongoing.

Chemiluminescence (CL) is the light emission from a chemical reaction. It is extensively used as a sensitive analytical method and has developed quickly since its introduction in the literature by Eilhardt Wiedemann in 1888.^17,18^ The most notable advantage of CL over traditional detection techniques is the great sensitivity achieved by eliminating the requirement for an external source of light. As a result, CL can minimize light scattering, increase detection sensitivity, and broaden the linear determination range.^19^ Since horseradish peroxidase (HRP) was initially proposed to catalyze luminol oxidation by H_2_O_2_ to greatly augment CL responses, different CL systems have been designed for the assay of diverse target samples.^20,21^ After luminol was discovered as a luminophore, numerous other CL luminophores, for instance, acridinium ester (lucigenin), 1,2-dioxetane, and their derivatives, were discovered.^22−24^ Recently, CL imaging technology has been appealing for *in vitro* as well as *in vivo* implementations due to the huge progress in the establishment of new CL platforms and novel optical detectors, for example, charge-coupled devices (CCD) and complementary metal-oxide-semiconductor (CMOS) image sensors.^25,26^ The technical progress in CCD and CMOS devices with great sensitivity and resolution has been a stimulus for their applications for CL imaging, and scientific grade CCD and CMOS detectors offer a high sensitivity for CL biosensing.^27^ Moreover, it is simple to monitor photon signals in microarrays to attain concurrent detection of multiple compounds with considerably decreased sample quantities. Consequently, CL imaging technology has been employed for recognizing diverse targets, *e.g.*, nucleic acids, enzymes, proteins, and organisms.^28−30^

On the one hand, poor tissue penetration and short timeliness of *in vivo* CL imaging models disabled the imaging of numerous key physiological and pathological processes in live systems, particularly in deep tissues.^31^ On the other hand, bioluminescence (BL) is common enough for living organisms and does not require the injection of chemicals; therefore, a BL imaging approach has become one of the most common noninvasive *in vivo* visualization technologies.^32,33^ In the absence of an excitation source, BL is produced by an enzymatic reaction that converts chemical energy into light in the living organism. It is simple to operate and most significantly has a high detection sensitivity. Also, the strong relationship between BL emission and metabolic processes assists in the data representativeness that allows experiment refinement, and as a result, a low number of animals is required for the study.^34^ Typically, luciferase/luciferin reaction is the most common BL system. So far, several luciferases have been utilized as new BL systems, and many of these BL systems have been successfully employed for cell and tissue imaging.^35,36^ Furthermore, BL imaging has been applied to observe cellular as well as intracellular motility, gene expression, and protein interactions in cells and tissues.^37−39^

As mentioned above, CL and BL are well-established photon-emission-based assay methods with the benefits of the absence of an external light source and thus eliminating photobleaching, background disturbance, and autofluorescence. As a result, these techniques have been developed effectively for the imaging of many significant analytes.^40,41^ Despite the extensive application of these imaging techniques, there are some areas that need urgent attention to further advance the techniques. For example, CL-based techniques are affected significantly by the experimental environments which have a significant impact on spatial resolution. The product of the enzymatic reaction, in particular, can diffuse in the solution before acting as a substrate for the CL reaction, resulting in a reduction in spatial resolution.^42^ Also, BL imaging approaches suffer from some problems, including the following: (1) Autoluminescence by substrates such as coelenterazine, in the absence of the enzyme, can also increase the luminescence background and cause variability. (2) Different half-lives of the used luciferases have different stability; (3) Cellular conditions, such as proteolytic degradation of the enzyme, pH, temperature, and H_2_O_2_ levels, are among many factors that could affect the BL signal.^43^ (4) Oxygen, hypoxia, and oxidative stress are limiting factors for all luciferase reactions; BL assays cannot be conducted under anaerobic conditions, and light emission from hypoxic tissues such as the bulk of a tumor is significantly suppressed.^44^ Also, the lack of oxygen could indirectly impact the BL reaction by affecting other cofactors needed for certain luciferase reactions (*e.g.*, ATP for Fluc).^45^ (5) Optical signal quenching is due to light absorption by pigmented molecules (*e.g.*, hemoglobin and melanin) and light scattering by mammalian tissues.^46^ (6) Light scattering decreases the spatial resolution of BL.^47^ This low resolution yields a poor localization of the BL source emanating from dispersed tumor cells and small metastatic areas.

Some review articles were reported on CL and BL imaging technologies; however, most of them focused either on the CL imaging assays or the *in vivo* BL imaging using common luciferase–luciferin pairs only.^28,34,48−50^ In the present review, the recent trends and applications (*in vivo* and *in vitro*) of both CL and BL imaging technologies using different CL and BL imaging systems are overviewed over the past five years, and the mechanisms of both CL and BL are described. Moreover, future perspectives on this remarkable topic are discussed.

## 2 General Mechanism of CL and BL Reactions

**Figure 1:**
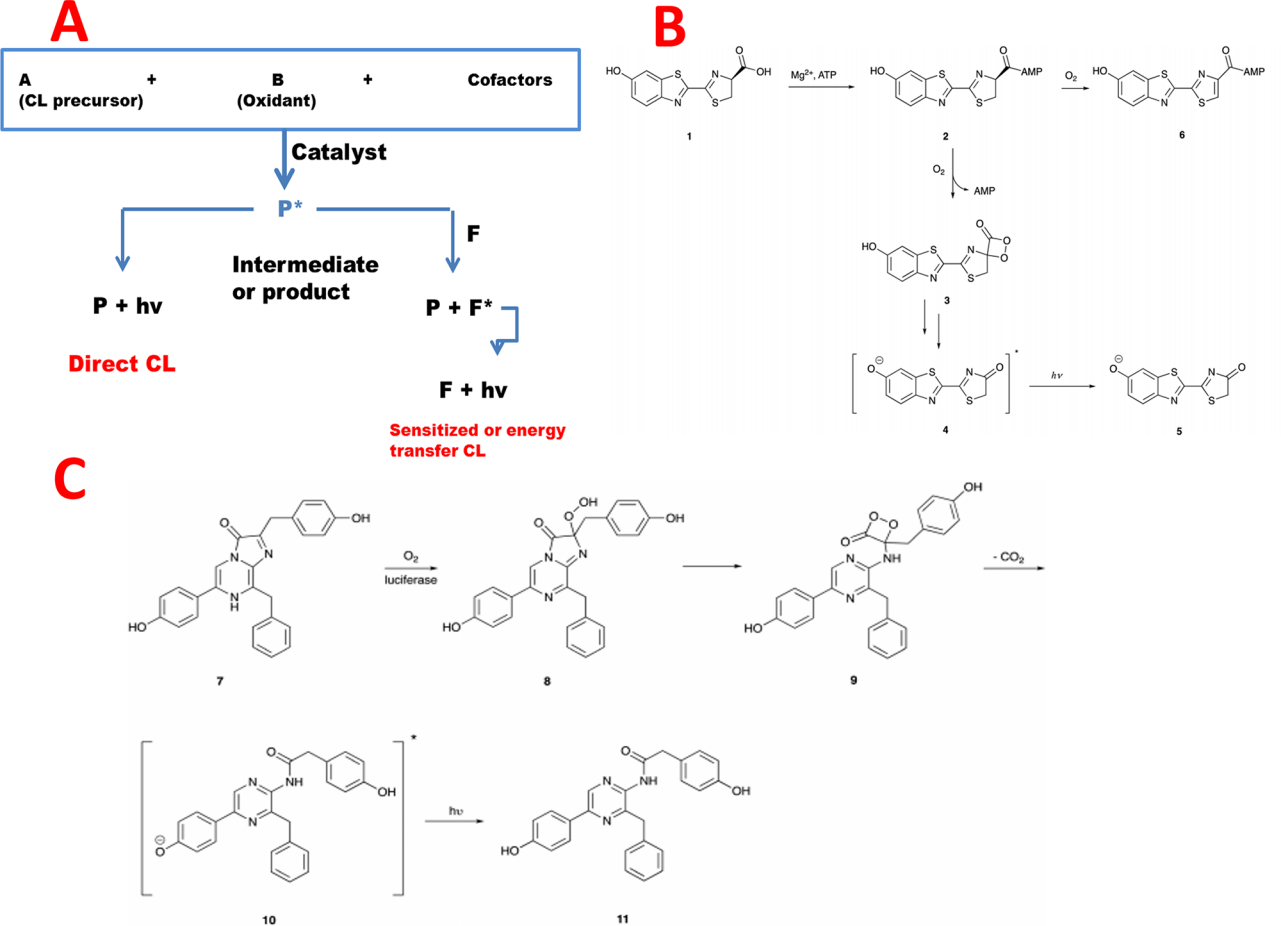
(A) Schematic illustrations of the different CL types (direct and indirect). The “*” represents excited products. Reprinted with permission from ref (53). Copyright 2021 MDPI. (B) The mechanism of firefly BL of d-luciferin bioluminescent reactions. Reprinted with permission from ref (58). Copyright 2021 Royal Society of Chemistry. (C) The mechanism of coelenterazine-based bioluminescent reactions. Reprinted with permission from ref (58). Copyright 2021 Royal Society of Chemistry.

CL is the light emission as a result of chemical reaction.^51^ Generally, the direct CL process can be mainly described in two stages. The first stage is the generation of an electronically excited intermediate (P*). The second stage is the emission of light by returning the excited state product, P*, to the ground state, P (Figure 1A, the left route).^28,52,53^ CL efficiency can be improved by using a sensitizer, F (Figure 1A, the right route), that acts as an energy acceptor that can take the energy from the initial excited intermediate, P*, and transform it into an emitter, F*. This process is also considered as sensitized or indirect CL.

BL is an attractive natural phenomenon by which living organisms generate and emit light. The BL reaction usually needs a luciferase, its luciferin substrate, and an oxidant which is frequently molecular oxygen. Some systems also demand energy in the form of ATP or NADH. One of the first luciferin structures discovered was d-luciferin which was discovered in fireflies and reported in the mid-1900s.^54^ Later, the luciferin coelenterazine and its luciferase were revealed in the deep-sea shrimp *Oplophorus gracilirostris*.^55^ As seen in Figure 1B, in the BL reaction involving d-luciferin, luciferase catalyzes the oxidation of d-luciferin **1** in two distinct steps. The first step is the activation of the carboxyl group of **1** through adenylation. The second step is the oxidation of the luciferyl adenylate (**2**) to produce oxyluciferin (**4**) as an electro-excited intermediate product through a dioxetanone intermediate **3**.^56^ The excited state oxyluciferin product **4** decays to its ground state **5** by emitting light, while the reactions involving coelenterazine, which is the substrate for around 15 different naturally occurring luciferases, proceed due to an enzymatically catalyzed oxidation reaction.^57^ Coelenterazine **7** is transformed to an excited state coelenteramide oxyluciferin (**10**) through a dioxetanone intermediate (**9**). The excited state of oxyluciferin returns to its ground state to emit a photon of blue light with a wavelength of 454–493 nm, dependent upon the enzyme (Figure 1C). This coelenterazine-based BL reaction is not based on ATP like the d-luciferin-based BL reaction.

## 3 CL Imaging Applications

Intracellular and intratissue imaging are critical for gaining a thorough understanding of gene expression, pathogenesis, and disease therapy. With the progress of the optical methods, CL has been well applied for the *in vitro* and *in vivo* visualization of different samples. Here, we will discuss the recent applications of different CL probes that have been employed for the *in vivo* and *in vitro* imaging of different targets (Table 1).

**Table 1:** Recent Applications of Different Reported CL-Based Imaging Probes

probe type	analyte	applications	ref
dioxetane-based CL probes	GGT	monitoring of GGT activity and its imaging *in vitro* and *in vivo* in mice models	(59)
	NK	*in vivo* quantification and imaging of NK cells activity against breast cancer cells in living mice	(60)
	HOCl	endogenous and exogenous visualization of HOCl in living mice	(61)
	biothiols	*in vivo* visualization of biothiols in tumor cells and tumor-bearing mice	(62)
	FAB	*in vitro* assay of FAB in plasma and its *in vivo* imaging in tumor cells of mice	(63)
CRET-based CL probes	ROS	real-time imaging of ROS in the peritoneal cavity as well as normal and cancerous tissues of mice	(64)
	physiological pH	imaging physiological pH in living animals	(66)
	amyloid beta (Aβ) species	imaging Aβ species in the brain	(67)
	hydrazine	exogenous (in a clean buffer environment) and endogenous (in living cells and mouse models) visualization of hydrazine	(68)
	ROS	real-time imaging of ROS and *in vivo* tracking of the tumor-targeted tissues	(65)
nanomaterials-based CL probes	ClO^–^	*in vivo* imaging and monitoring of the endogenously generated ClO^–^ in living cells	(69)
	PSA	detection and imaging of PSA in human serum samples	(70)
	ALP	diagnosis of liver cancer and its treatment without applying external light radiation	(71)
	ClO^–^	*in vivo* imaging of the biologically formed ClO^–^ from malignant tissues in living animals	(72)
near-infrared (NIR)-based CL probes	formaldehyde	imaging of formaldehyde in the living cells of mice	(73)
	RONS	imaging of the reactive oxygen and nitrogen species (RONS) in the kidneys of living mice	(74)
	ONOO^–^ species and β-galactosidase	assay and imaging of ONOO^–^ species and β-galactosidase in cells of a tumor animal model	(75)

### 3.1 Dioxetane-Based CL Probes

**Figure 2:**
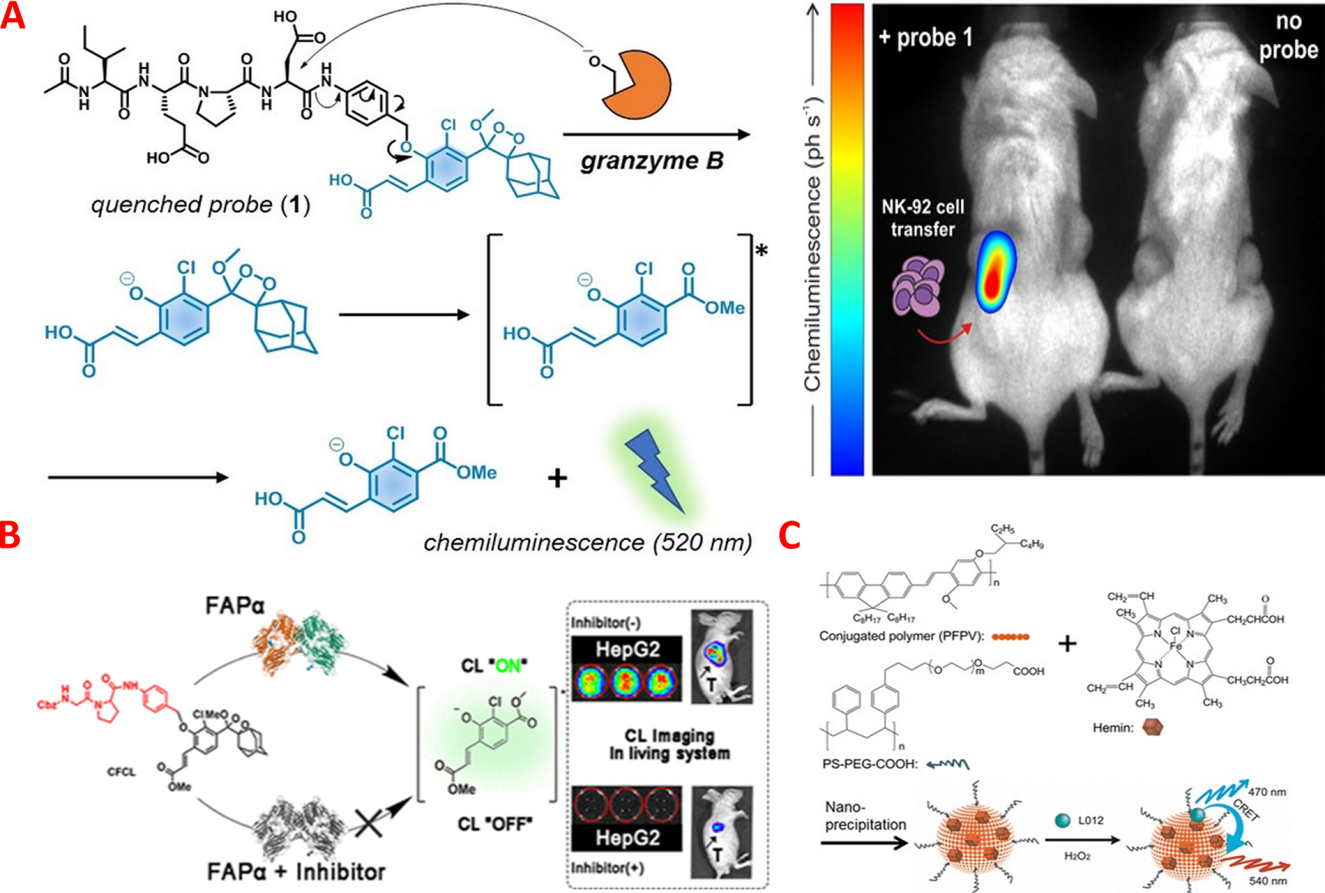
(A) Proposed activation mechanism of the CL probe for the *in vivo* detection and observing NK cell activity and CL images of the adoptively transferred NK-92 cells in mice. Reprinted with permission from ref (60). Copyright 2021 Wiley-VCH. (B) Diagram representation of the developed CL probe for the quantification and imaging of FAPα. Reprinted from ref (63). Copyright 2021 American Chemical Society. (C) H-Pdots-based CL probe and its principle of the catalytic CL mode. Reprinted from ref (64). Copyright 2018 American Chemical Society.

Dioxetane scaffold-based CL probes have been widely used for different imaging applications; for example, An **et al.** constructed a γ-glutamyl transpeptidase (GGT) “signal-on”-type CL platform for monitoring GGT activity and its imaging *in vitro* and *in vivo* in mice models.^59^ The idea of the developed probe was dependent on using a modified Schaap’s phenoxy-dioxetane with an electron-withdrawing acrylic group (specific recognizing moiety for GGT) and *p*-aminobenzyl alcohol as a self-immolative linker. With such a structure, the probe exhibits no CL intensity, but upon the addition of GGT, the probe reacts with it and strong CL is attained in an aqueous solution, enabling a specific estimation of GGT with high sensitivity. The established CL approach has been utilized for the detection of GGT elevated levels in lipopolysaccharide-treated mouse serum. Furthermore, CL imaging of GGT activity was achieved in GGT-positive tumors in living mice after intravenous administration. This work indicates the great potential of a GGT-signal-on CL probe for plasma tests and molecular imaging, which might be useful in the discovery of GGT-associated disorders. Moreover, Scott and co-workers established a novel turn-on CL probe enclosing a granzyme B-reactive peptide substrate attached to a phenoxydioxetane scaffold for the *in vivo* quantification and imaging of natural killer (NK) cell activity against breast cancer cells in living mice (Figure 2A).^60^ Regarding rapidity and sensitivity, the established CL probe surpasses the available marketable fluorogenic reagent Ac-IEPD-AMC and may be employed to quantify NK cell activity in cocultures of human NK cells and cancer cells. Additionally, the developed probe has been employed in a preclinical mouse model of NK cell adoptive transfer to observe the tumors that can be recognized by NK cells using the *in vivo* CL imaging by the established probe. The work shows how “signal-on”-type CL probes may be used to assess the effectiveness of NK cell-based immunotherapies in live tumors. Also, Wang and co-authors established a novel “signal-on” CL biosensor for the selective monitoring of hypochlorous acid (HOCl) *in vivo* and *in vitro* by a combination of an identification moiety called 4-aminophenyl ether with the adamantylidene-dioxetane luminophore unit.^61^ Notably, HOCl could cleave the identification moiety efficiently, resulting in a significant “signal-on” CL with a LOD of 10 nM. The developed CL platform has been employed for the endogenous and exogenous visualization of HOCl in living mice. Furthermore, they were able to distinguish tumor zones from normal cells by monitoring the anomalous levels of HOCl in cancer locations. For bioimaging biothiols, Fu *et al.* developed two turn-on CL probes (probe I and probe II) and examined their functions for biological thiols imaging in cells.^62^ The developed probes were based on using a biothiol recognition group (2,4-dinitrobenzenesulfonyl) for biothiols detection. This group was directly attached to the OH moiety of phenoxy-dioxetane luminophore. Probes I and II are varied in their phenol-dioxetane substituents. Probe I contains *o-*chlorine and probe II contains *p-*hydroxymethyl. Two probes produced considerable “signal-on” CL response upon glutathione (GSH) cleavage. However, the intensity of CL response based on probe II decreased when GSH concentration increased over 5 mM, but probe I demonstrated substantially greater CL response and wider linearity (0.5–50 mM), making it highly appropriate for detecting biological thiols. Chlorine substitution in probe I played a significant function in the imaging process due to the impact of halogen which offers a lower p*K*_a_ value and essential improvement of the CL signal intensity. The designed probe I was employed for the *in vivo* visualization of biothiols in tumor cells as well as in tumor-bearing mice. Fibroblast activation protein-alpha (FAPα) is a major regulator of the microenvironment in a variety of diseases and is emerging as a cancer biomarker for cancer diagnosis and therapy. Recently, Fu *et al.* developed the first CL platform for FAPα *in vitro* assay in plasma and its *in vivo* imaging in tumor cells of mice (Figure 2B).^63^ The developed probes were based on using glycine-proline dipeptide ligand as FAPα-specific moiety in the adamantylidene-dioxetane molecule. As a result, they constructed three different CL probes (probe I, probe II, and probe III) with the dipeptide ligand obstructed by N-terminal benzyloxycarbonyl, *N*-*tert*-butoxycarbonyl, or *N*-quinoline-4-carboxylic acid, which served as a masking moiety to limit the CL emission. Molecular docking simulation revealed that probe I has the best specificity for distinguishing FAPα from dipeptidase IV and prolyl oligopeptidase. Upon the addition of FAPα to probe I, a high CL intensity was observed, thus probe I has been used for the specific estimation of FAPα with a LOD of 0.785 ng/mL. Moreover, probe I has been applied for *in vitro* determination of the target analyte in serum and tissue samples and its *in vivo* imaging in tumor cells of mice. Additionally, this innovative assay method might be simply expanded to assess FAPα inhibitors as seen in Figure 2B. Overall, the proposed probe I will provide a simple and cost-effective option for early pathology identification and drug therapy screening.

### 3.2 Chemiluminescence Resonance Energy Transfer (CRET)-Based CL Probes

**Figure 3:**
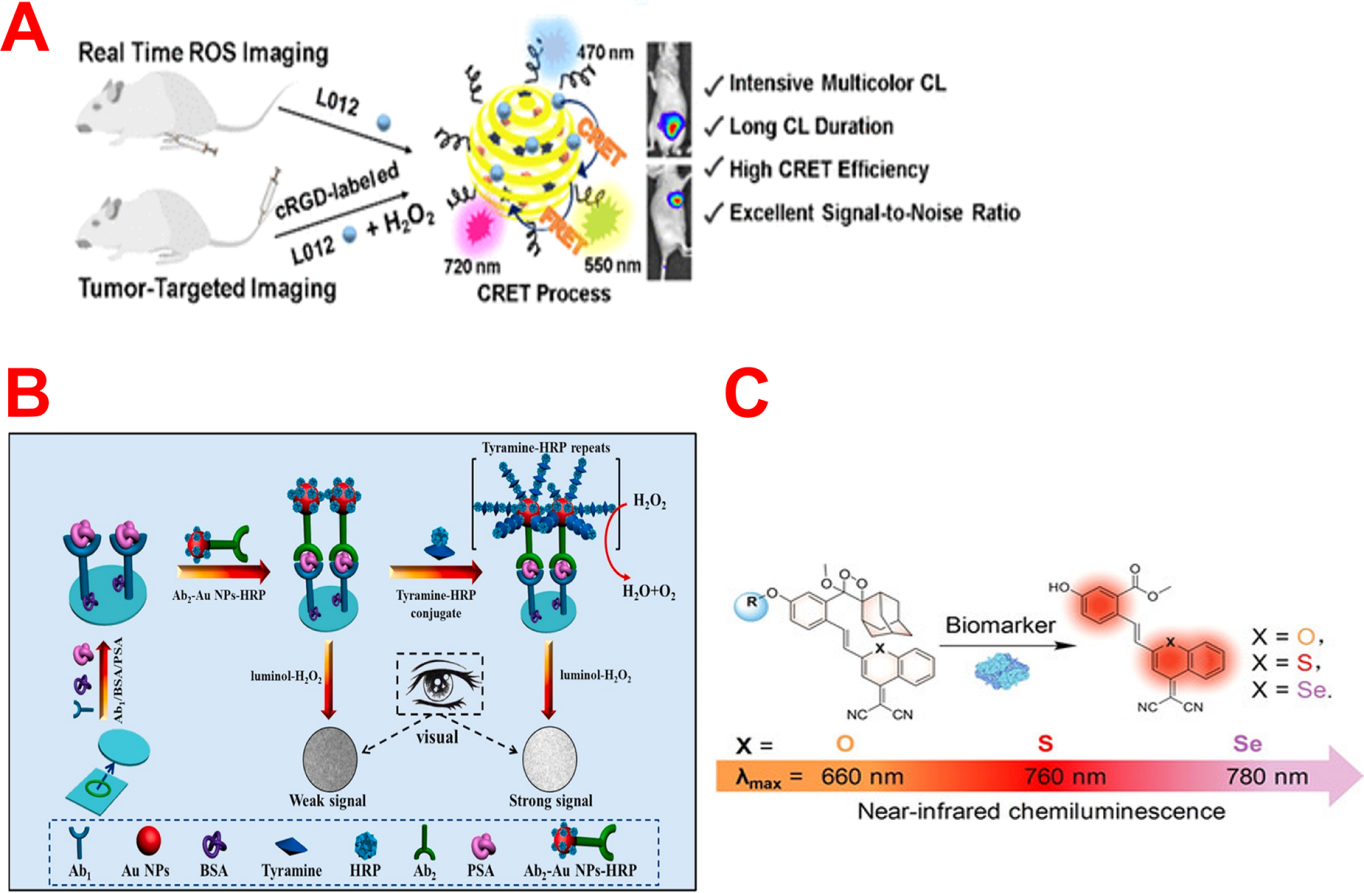
(A) CRET-Pdots-based CL probe with multicolor CL and other features for imaging of ROS. Reprinted from ref (65). Copyright 2021 American Chemical Society. (B) Diagram illustration of the construction steps of the developed PSA CL immunosensing platform. Reprinted with permission from ref (70). Copyright 2021 Elsevier. (C) NIR-based CL probes for imaging of ONOO– species and β-galactosidase. Reprinted with permission from ref (75). Copyright 2021 Wiley-VCH.

Cai *et al.* proposed a CRET-based probe from polymer dots (Pdots) and hemin-Pdots (H-Pdots).^64^ H-Pdots consist of hemin and fluorescent conjugated polymer. The H-Pdots were fabricated by utilizing the nanoprecipitation approach, as seen in Figure 2C. In the existence of CL substrates and hydrogen peroxide, H-Pdots display a strong CL response and long-lasting emission over 10 h. They used L012 for checking the catalytic activity of H-Pdots on the reaction of L012 and H_2_O_2_. Notably, H-Pdots have strong catalytic activity, provide a red shift of CL emission wavelength, display strong stability, high biocompatibility, and great responsiveness to reactive oxygen species (ROS). H-Pdots have been used for the real-time visualization of ROS concentrations in the peritoneal cavity as well as normal and cancerous tissues of mice. Another CRET-Pdots-based CL probe was developed by Li *et al.* utilizing catalytic CL substances as energy donors as well as fluorescent polymers and dyes as energy acceptors for the *in vivo* imaging of ROS (Figure 3A).^65^ Fe(III) deuteroporphyrin IX as a CL catalyst, fluorescent polymers, and dyes were used for the fabrication of CRET-Pdots. The CL signal amplitudes as well as duration were significantly improved by utilizing the CL reaction of luminol analogue with H_2_O_2_. Moreover, CRET-Pdots displayed great multicolor CL, persistent CL time (over 8 h), a tunable emission wavelength (470–720 nm), and significant CRET effectiveness (50%). The developed Pdots have been effectively employed for the real-time visualization of ROS and *in vivo* tracking of the tumor-targeted tissues.

Biological pH regulation is essential for the optimal functioning of the whole body and cells, and changes in physiological pH can lead to several diseases. In light of this, Ryan *et al.* developed the ratio-pH CRET probe, which is composed of a pH-sensitive carbofluorescein fluorophore that is attached to an acrylamide 1,2-dioxetane chemiluminescent scaffold.^66^ The established CRET probe accurately measures pH change between 6.8 and 8.4, enabling it to be a useful method for detecting biological pH. The developed probe has been used as the first single-molecule CRET platform for imaging physiological pH in living animals.

Recently, Yang and co-workers fabricated a novel “signal-on”-type CL probe for imaging amyloid beta (Aβ) species in the brain.^67^ They preferred to use imidazo[1,2-*a*]pyrazin-3(7*H*)-one as the scaffold, rather than other CL scaffolds (lucigenin, dioxetane, and luminol) owing to its flexibility to be chemically modified for emission wavelength range extension and excellent stability. The established probe can successfully pass the blood–brain barrier (BBB) and offers good contrast for AB plaques as well as cerebral amyloid angiopathy. The obtained findings of the *in vivo* brain imaging reveal that the CL signals of the probe from 5-month-old transgenic 5xFAD mice are 1.80-fold greater than that of age-matched wild-type mice. Furthermore, the study revealed that it is possible to promote a dually amplified signal (DAS) *via* CRET (DAS-CRET) using two nonconjugated smart probes (the developed probe and CRANAD-3) in brain homogenates, solutions, and *in vivo* whole brain imaging. The findings reveal that DAS-CRET can offer a 2.25-fold signal enhancement between 5-month-old 5xFAD mice and wild-type mice.

Moreover, Li and colleagues developed an innovative photoactivatable red CL AIEgen platform (ACL) for the exogenous (in a clean buffer environment) and endogenous (in living cells and mouse models) visualization of hydrazine.^68^ The developed CL probe relied on a combination of the red-emission AIEgen fluorophore (TPEDC), which has a superior ^1^O_2_ production capability, and the dioxetane precursor unit for the estimation of hydrazine *in vitro* and *in vivo*. By applying white light irradiation, the C=C bond attached to adamantine unit in ACL was first transformed to dioxetane to form the activated ACL probe. Then, the self-immolative interaction was prompted upon the removal of the acylated phenolic hydroxyl moiety in ACL by hydrazine target analyte, causing the release of the high energy kept in the 1,2-dioxetanes, and then, the chemiexcitation was prompted, generating red CL through the intramolecular CRET from dioxetane to TPEDC.

### 3.3 Nanomaterials-Based CL Probes

Duan’s group fabricated a novel CL probe for imaging the endogenously produced hypochlorite (ClO^–^) in a living mouse model of peritonitis inflammation.^69^ In this work, the established CL platform was dependent on the direct oxidation of conjugated polymers (CPs) found as a backbone in the fabricated polymer nanoparticles (CPNs) by hypochlorite. Vinylene bond (C=C) found in polyfluorene-vinylene (PFV)/polyphenylenevinylene (PPV) could be oxidized by ClO^–^ to generate an unstable PFV- or PPV-dioxetane product that can decompose spontaneously into PFV- or PPV-aldehyde and emit bright CL intensity. The developed probe has been used for the detection of ClO^–^ from 2 to 30 μM and was employed for *in vivo* imaging and monitoring of the endogenously generated ClO^–^ in living cells.

In 2021, Liu’s group designed a novel CL imaging immunosensor with several signal-enhancing capabilities for prostate-specific antigen (PSA) determination and imaging.^70^ To establish this platform, they fixed the primary PSA antibody on an O-ring zone of a glass slide for identifying the target analyte. Then they synthesized gold nanoparticles (AuNPs) and functionalized them with an Ab2 and HRP. The outstanding catalytic impact of AuNPs and HRP within the HRP-AuNPs-Ab2 nanocomposite on the classical CL system (luminol-H_2_O_2_) offered dual-signal augmentation for PSA determination (Figure 3B). For improving the probe’s sensitivity, a tyramine (TA) signal augmentation (TAA) approach was implemented. Upon the addition of TA-HRP conjugates and H_2_O_2_, much TA-HRP was attached onto the HRP-AuNPs-Ab2 nanocomposite, resulting in multiple signal augmentation due to the high concentration of HRP on the detection area. The CL platform sensitivity was enhanced by the synergistic behavior of HRP-Au NPs-Ab2 and the TAA approach. The PSA was quantified with PMT and visually detected by CCD with detection limits of 0.05 and 0.1 pg/mL, respectively.

An important therapeutic technique called photodynamic therapy (PDT) has been widely used in cancer therapy. However, PDT suffers from some limitations. For example, an external light source may not have adequate penetration depth and PDT may cause damage to nearby normal healthy tissues. Hence, the scientists designed a CL theranostics platform for avoiding PDT limitations. For example, Fan *et al.* developed a CL theranostics platform abbreviated as “MSN@H6L@β-CD@AMPPD NPs” for the specific diagnosis of liver cancer and its treatment without applying external light radiation.^71^ The established probe relied on the host–guest interaction of 3-[(2-spiroadamatane)-4-methoxy-4-(3-phosphoryloxy)-phenyl-1,2-dioxetane] dioxetane with β-cyclodextrin attached to the mesoporous silica nanoparticles (MSNs) functionalized with (4-carboxyphenyl) porphyrin. The liver cancer biomarker alkaline phosphatase can specifically hydrolyze these NPs, enabling liver cancer-targeting CL. Recently, the hypochlorite anion (ClO^–^) as a significant colonic cancer marker was detected and visualized by the development of a new CL probe.^72^ Li and co-workers fabricated poly[(9,9-di(2-ethylhexyl)-9*H*-fluorene-2,7-vinylene)-*co*-(1-methoxy-4-(2-ethylhexyloxy)-2,5-phenylenevinylene)] (PFV-*co*-MEHPV, namely, CP1), and then it was captured into MSNs. After that, the as-prepared nanoparticles were premodified with polyphenylenevinylene (PPV) *viain situ* polymerization to make bright PPV@MSN-CP1 nanoparticles. Notably, ClO^–^ could oxidize the vinylidene bond of the developed probe to produce PPV-dioxetane intermediate products emitting light. PPV@MSN-CP1 exhibited excellent specificity for ClO^–^ determination in the presence of different biological oxidants. The dynamic range of the developed method was 4–90 μM, and the LOD was 1.02 μM. Moreover, it was effectively employed for *in vivo* visualization of the biologically formed ClO^–^ from malignant tissues in living animals.

### 3.4 Near-Infrared (NIR) CL Probes

Bruemmer and co-authors developed new CL probes (CFAP540 and CFAP700) for the visible (540 nm) and NIR (700 nm) determination and imaging of formaldehyde in the living cells of mice.^73^ Formaldehyde release resulting from endogenous folate metabolism can be observed using the CFAP700 CL probe, paving the way for the use of CFAPs to investigate formaldehyde physiology and pathophysiology. The linear range of formaldehyde detection using CFAP540 and CFAP700 was 25–50 μM. The CFAP700 probe was effectively utilized for tracking the exogenous formaldehyde addition in living animals in a dose-dependent manner and endogenous formaldehyde release from the folate cycle, mainly through tetrahydrofolate metabolism.

Two NIR chemiluminescent reporters (NCRs) for observing reactive oxygen as well as nitrogen species (RONS) in the kidneys were fabricated by Huang *et al.*(74) The molecular design enables NCRs to easily go to the kidneys of living mice, selectively interact with their RONS indicators, and reflect oxidative/nitrosative stress in the kidneys following nephrotoxicity exposure. NCR1 (for ROS) and NCR2 (For RONS) chemiluminescence half-lives were estimated to be 7.6 and 9.1 min, respectively, which was sufficient for live imaging. Two reporters were investigated for visualizing RONS in HK2 proximal tubule epithelial cells. Cisplatin (an anticancer chemotherapeutic medication) was applied to the cells at a toxic dose for the indication of nephrotoxicity. Also, they compared NCRs with the green chemiluminescent counterparts (GCR) regarding the penetration depths and the imaging sensitivity of both. They found that NIR CL of NCR1 was detectable up to the tissue depth of 2 cm; however, the green chemiluminescence of GCR1 was just measurable at 1.5 cm. Moreover, the detection sensitivity for NCR1 was higher than GCR1 reporter. These findings revealed that NCRs had greater tissue penetration depth and better sensitivity than known green reporters.

Recently, Huang *et al.* constructed two CL probes for the selective assay and visualization of ONOO^–^ species and β-galactosidase (Figure 3C).^75^ Two chemiluminophores have demonstrated long NIR emissions (>750 nm). They were fabricated by linking dicyanomethylene-4*H*-benzothiopyran (probe I) or dicyanomethylene-4*H*-benzoselenopyran (probe II) with a dioxetane molecule. Attaching the CL luminophores with various cleavable groups results in NIR CL biosensors with deep tissue penetration depths (2 cm) that only emit responses upon reactivity with ONOO^–^ and β-galactosidase. Furthermore, probe II was verified in cells in a tumor animal model, demonstrating its capacity to quantify β-galactosidase and distinguish its expression points in various cells. As a result, this work presents an atomic alternation strategy for developing NIR chemiluminophores that may be used as universal scaffolds for tracking different significant analytes.

Notably, most of the CL probes are based on 1,2-dioxetane derivatives as turn-on platforms for various substrates; however, their synthetic procedures are difficult and therefore hinder their large-scale utilization. In contrast, CRET-based probes derived from CL Pdots are easily prepared, have intensive multicolor emissions, and are characterized by their long-lasting emission, making them promising alternatives.^64^ NIR-based CL probes which are characterized by their deeper penetration capacity will be used as universal scaffolds for tracking different significant analytes in deep tissues.

## 4 BL Imaging Applications

BL is a luminescence process created by living organisms in which energy is generated by enzymatic processes rather than an external light source.^33,76^ Various luciferases from beetles and undersea luminous animals were used for BL imaging by sequencing and cloning.^77,78^*In vivo* BL imaging involves the introduction of luciferase into animals or the integration of the luciferase gene into the cell chromosome, which subsequently catalyzes the luciferin substrate to produce BL. There is no need for external light in the BL process, and emission is a result of own chemicals recombination of a living cell.^79^ Owing to their great biocompatibility, sensitivity, and selectivity, BL-based imaging platforms are currently broadly employed in medical research, life sciences, and drug development to investigate tumor metastasis,^80^ cell death,^81^ bacterial and viral infection,^82,83^ protein interactions, and transgenes.^84,85^ Moreover, to boost imaging sensitivity, various new probes, for instance, upconversion nanoparticles and quantum dots, have been coupled with conventional luciferase systems.^86,87^Table 2 summarizes the recent applications of different BL probes that have been used for the imaging of different analytes.

**Table 2:** Summary of BL Probes for Imaging Different Analytes from the Years 2018 to 2022

probe	analyte	applications	ref
BL probes for metal ions detection	Co^2+^	estimation and imaging of the accumulation and fluctuation of Co^2+^ level in a mouse model	(88)
	Hg^2+^	*in vitro* detection of Hg^2+^ in cell culture and *in vivo* imaging of Hg^2+^ using transgenic FVB-luc+ mice	(89)
	Fe^2+^	tracking extracellular Fe^2+^ amounts in live cells	(90)
	Pd^2+^	imaging the variations in the concentrations of Pd^2+^ in living cells and animals	(91)
BL probes for the detection of different amino acids	Cys	*in vivo* imaging of Cys	(92)
	Cys	detection of the exogenous and endogenous Cys and imaging Cys in the whole animal	(93)
	biothiols	monitoring of biothiols in African green monkey fibroblast COS-7 cells and human breast adenocarcinoma MCF-7 cells	(94)
	biothiols	imaging of biothiols in living cells	(95)
	Sec	imaging the Sec in living cells and MCF-7-Luc tumor of mice	(96)
BL probes for the detection of reactive species	superoxide anion radicals	estimation of superoxide anion radicals (O_2_˙^–^) and its imaging in the Huh7 cells	(97)
	superoxide anion radicals	real-time observation of O_2_^•–^ in both cancer cells and tumors	(98)
	ONOO^–^	imaging of intracellular ONOO^–^ in living cells and mice	(99)
BL probes for the detection proteins	uPA	tracking of uPA activity in cancerous tissues of living mice	(100)
	FAB	imaging of FAP levels in living cells and in living mice	(101)
	FAB	*in vivo* imaging of FAP of fluc-transfected 4T1 tumor-bearing mice	(102)
	FAB	endogenous imaging of FAP in living cells and nude mice bearing MGC-803-luc tumors	(103)
	NTR	*in vitro* and *in vivo* imaging of NTR in hypoxic cancers	(104)
	reelin	imaging of reelin in the HEK293T cells	(105)
BL probes for the detection of different enzymes activities	MMP-14 activity	imaging of MMP-14 activity in tumor-bearing mice	(106)
	MMP-2 and MMP-9 acticities	imaging of MMP 2,9 after ethanol-induced corneal injury of mice	(107)
	GGT activity	imaging of the endogenous GGT activity in mice	(108)
	pantetheinase activity	imaging of the endogenous pantetheinase activity using pathogen-free luciferase-expressing transgenic mice	(109)
	CB	imaging of CB in transfected MDA-MB-231 tumor-bearing nude mice	(110)
	ES and HAC activities	imaging of ES and HAC activities in nude mice bearing the fLuc-transfected MDA-MB-231 tumor	(111)
	sulfite oxidase deficiency	*in vivo* visualization of sulfite levels in living animal	(112)
	hyaluronidase 1 (Hyal-1) activity	imaging the Hyal-1 alterations in living cells and animals	(113)
	ALP and GGT	imaging of the co-overexpressed ALP and GGT in fLuc-231 breast cancer cells	(114)
	TYR	imaging of TYR activity of mice xenografted with Luc-transfected B16 cells tumors	(115)
other BL probes	carbon monoxide (CO)	imaging of CO of nude mice xenografted with luciferase-transfected Huh7 tumors	(116)
	CO	imaging of CO of nude mice xenografted with luciferase-transfected Huh7 tumors	(117)
	human mesenchymal stem cells (hMSCs)	*in vivo* tracking of the living hMSCs	(118)
	NE	noninvasive imaging and real-time monitoring of NE in living animals	(119)
	H_2_S	imaging of H_2_S of malignant cells and nude mice	(120)
	polysulfides	imaging the fluctuations in H_2_S_*n*_ level in living cells	(121)

### 4.1 BL Probes for Metal Ion Detection

**Figure 4:**
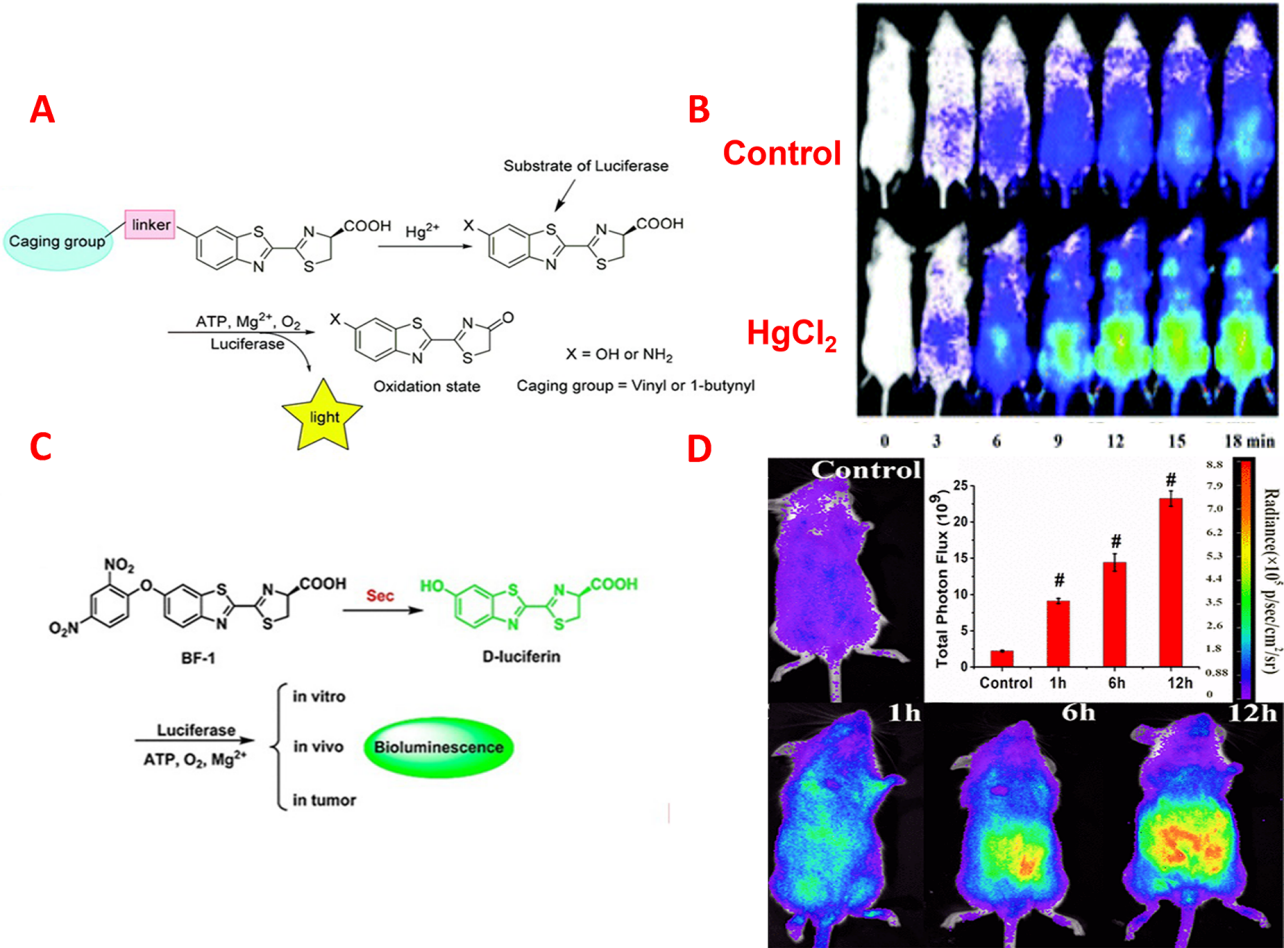
(A) Assay of Hg^2+^ by using BL probes relied on the luciferin/luciferase system. Reprinted with permission from ref (89). Copyright 2018 Royal Society of Chemistry. (B) BL imaging of FVB-luc^+^ mice injected with the developed probe and Hg^2+^. Reprinted with permission from ref (89). Copyright 2018 Royal Society of Chemistry. (C) Diagram illustration of the proposed reaction response of the established BL probe for Sec. Reprinted from ref (96). Copyright 2019 American Chemical Society. (D) BL images for imaging Sec in FVB-luc^+^ mice. Reprinted from ref (96). Copyright 2019 American Chemical Society.

Ke *et al.* developed a “signal-on”-type BL platform for detecting and imaging cobalt ion accumulation in a mouse model.^88^ The developed BL probe was synthesized by attaching a tetradentate ligand N_3_O to the 6′-OH moiety of luciferin. Notably, Co^2+^ could selectively react with the developed probe in the presence of oxygen, resulting in the release of the d-luciferin molecules and consequently resulting in an intense BL signal response. The developed BL probe was utilized for the estimation of the accumulation and fluctuation of Co^2+^ level in a mouse model. Also, Ke *et al.* designed a new BL probe for the noninvasive visualization of mercury ions and imaging of their accumulation levels within living animals (Figure 4A).^89^ The developed probe was based on using the oxymercuration–reduction interaction that converts an alkene/alkyne into the corresponding alcohol/ketone. They have used vinyl moiety for blocking the active site of luciferin. In the presence of Hg^2+^, a selective reaction between the developed probe and Hg^2+^ occurred and resulted in the release of luciferin which acts as an active substrate for luciferase for giving high BL response. The relative BL intensity of the developed probe with different anionic interfering species has been investigated. The obtained results indicated that the method had excellent selectivity for the detection of Hg^2+^. The developed probe has been used for the *in vitro* detection of Hg^2+^ from 0 to 100 μM in cell culture. Moreover, the developed probe has been used for *in vivo* imaging of Hg^2+^ using transgenic FVB-luc^+^ mice (Figure 4B).

Iron is a necessary element for biological systems. Feng *et al.* developed a novel BL probe providing a powerful approach for detecting Fe^2+^ exogenously and endogenously.^90^ The design plan of the developed approach relied on the insertion of a nonheme 2-His-1-carboxylate ligand group into the luciferin unit to prevent substrate-enzyme interaction. In the presence of Fe^2+^, substrate-luciferase interaction occurred and a noticeable enhancement of light intensity (13 times) was obtained in 100 min. The established probe has an iron-responsive reaction that allows for a quick BL signal intensity increase and a target-specific readout. The authors demonstrated the efficacy of probe by detecting Fe^2+^ levels in an aqueous buffer with high specificity over different interfering metal ionic species and tracking extracellular Fe^2+^ amounts within live cells. Furthermore, the obtained results in the CLP animal model indicate its usability in the investigation of the changes in endogenous iron levels.

Other innovative BL probes for palladium(II) have been proposed by Ke and co-authors relying on a reactivity-based strategy.^91^ They have caged the luciferin by using propargyl ether or propargyloxy carbonyl groups for designing probes I and II, respectively. The proposed probes displayed high sensitivity (LOD = 0.5 μM) and reasonable discrimination toward Pd^2+^*in vitro*. Specifically, probe II was found as a feasible molecule capable of imaging the variations of Pd^2+^ concentrations in living cells and animals, offering a helpful approach for tracking Pd^2+^ in biological samples.

### 4.2 BL Probes for the Detection of Amino Acids

Zhang and co-workers synthesized a “signal-on” BL system for the selective assay and *in vivo* visualization of cysteine (Cys).^92^ The established BL probe was based on blocking the OH moiety of d-luciferin by the acrylic ester group which is well-known as a specific recognition group for Cys detection. Michael addition of Cys to acrylate results in the formation of d-luciferin which is used for BL imaging. The proposed approach has been used for quantification of the target amino acid with a LOD of 88 nM *in vitro*, and in living mice, it was also successfully visualized.

Another BL approach for the estimation and imaging of Cys in living cells was established by Hu *et al.*(93) The developed probe relied on the attaching of an acryloyl group into the luciferin scaffold to hinder the enzyme–substrate interaction and offers high selectivity of the proposed probe toward Cys. Upon the addition of Cys, a condensation reaction of acrylate with Cys will occur and luciferin will be produced which is then catalyzed by a luciferase enzyme in the presence of ATP, Mg^2+^, and oxygen to generate a strong BL response. The established “signal-on” BL probe has been used for estimating exogenous and endogenous Cys and for imaging Cys in the whole animal.

Furthermore, Suzuki’s group established a chemoselective probe for the assay of thiols in fluorescence as well as BL modes.^94^ The synthesized probe relied on using sulfinate ester luciferin to give a chemoselective probe displaying a ratiometric and “signal-on”-type behavior against thiols in fluorescence and BL modes, respectively. The constructed probe has demonstrated great selectivity, a high S/N ratio (>240 for BL), and a detection limit relevant to physiology (80 nM for Cys), all of which are appropriate characteristics for a sensitive BL approach. The developed probe was then used to identify the changes in thiol levels caused by oxidative stress in a BL test including African green monkey fibroblast COS-7 cells as well as human breast adenocarcinoma MCF-7 cells.

Another biothiol-targeting BL probe has been synthesized and used for the selective assay of biothiols and their *in vivo* observation in living systems.^95^ For this purpose, the authors developed a coelenterazine-based biothiol platform by incorporating an acryloyl moiety into a coelenterazine derivative. The developed probe has been characterized by its distinctive merits such as high sensitivity, rapidity, and selective “signal-on” behavior toward three biothiols (Cys, GSH, and homocysteine (Hcy)) in aqueous conditions. Also, it has a low cytotoxicity that allows the effective application for visualizing the variations in biothiol concentrations in living cells. Moreover, the developed probe was integrated with a NIR fluorescent protein (iRFP713)-fused luciferase to spread the BL emission wavelength range into the NIR area.

Zhang *et al.* fabricated the first BL biosensor for real-time estimating of selenocysteine (Sec) *in vivo*.^96^ As seen in Figure 4C, the synthesized probe was based on caging the 6′-OH moiety of d-luciferin substrate with a 2,4-dinitrophenyl ether unit. Sec can selectively cleave this moiety to release luciferin, which is then recognized by firefly luciferase, resulting in the emission of a BL signal. The established BL probe was effectively used for the quantification of Sec with a low LOD of 8 nM and for visualizing the target analyte in living cells as well as in mice. Moreover, BL photos of nude mice having MCF-7-Luc tumors confirmed that the established probe could be effectively applied for investigating the Sec levels variations in tumorous tissues (Figure 4D).

### 4.3 BL Probes for the Detection of Reactive Species

Lu’s group established a new BL probe for the estimation of superoxide anion radicals (O_2_˙^–^) and their imaging in the cells.^97^ They blocked the OH moiety of d-luciferin substrate by using a phosphine moiety for developing a phosphinate-based BL approach. They used the phosphinate-based probe for the specific quantification of O_2_˙^–^ at the nanomole level and the probe has shown excellent selectivity toward O_2_˙^–^ in the presence of other ROS. The probe was also examined to be nontoxic to living cells and was effectively used for detecting endogenous O_2_˙^–^ in Huh7 cells utilizing phorbol-12-myristate-13-acetate (PMA) and monodisperse polystyrene particles as conventional and novel O_2_˙^–^ stimulators, respectively. This is the first small-molecule phosphinate-based BL probe, which will allow researchers to unlock the mystery of O_2_˙^–^ in human health and illness.

**Figure 5:**
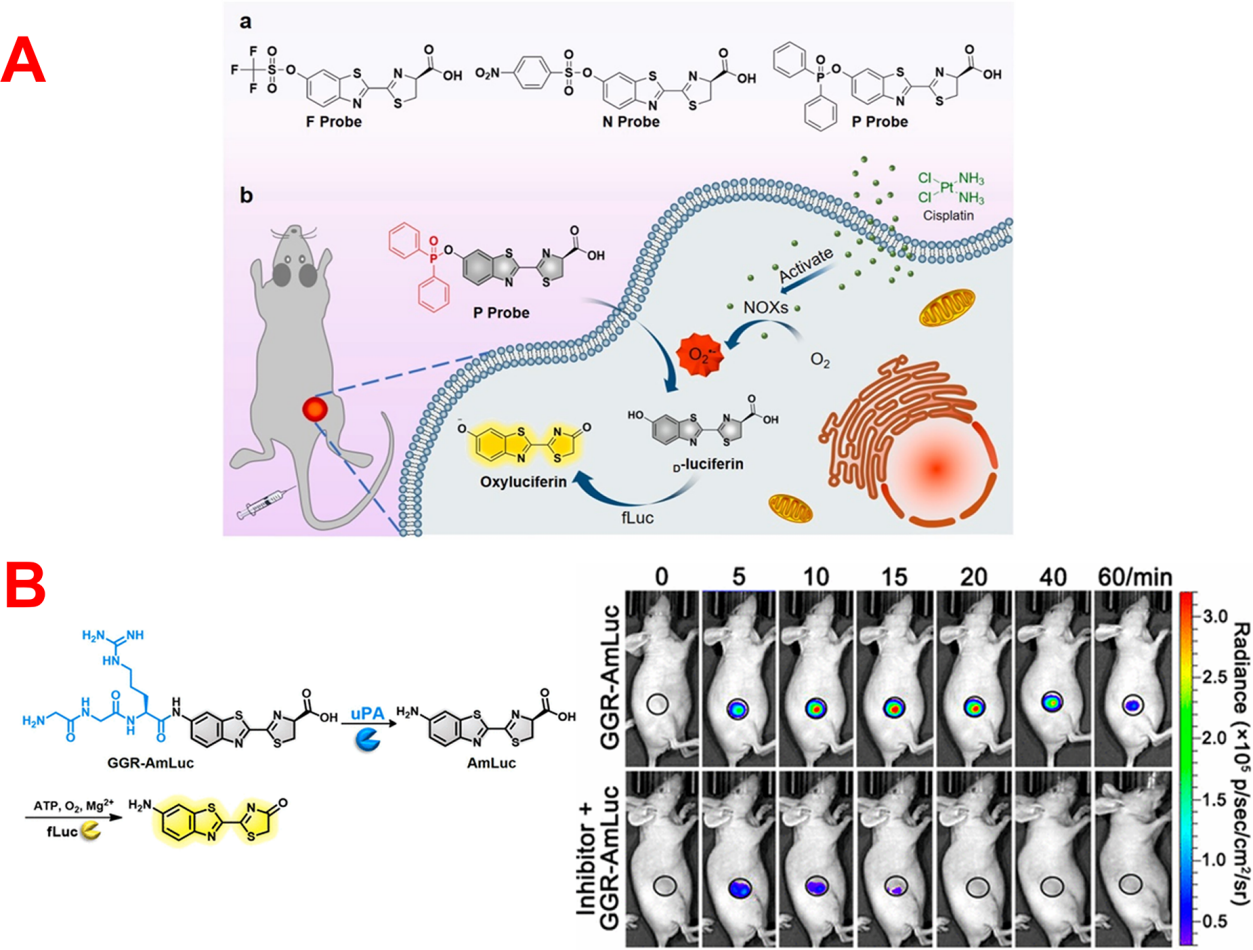
(A) Schematic illustration of the construction of three different BL probes for visualization of O_2_^•–^. Reprinted with permission from ref (98). Copyright 2022 Elsevier. (B) Diagram illustration of uPA-triggered BL generation of the developed GGR-AmLuc BL probe and BL imaging of uPA activity in experimental and control groups of mice. Reprinted from ref (100). Copyright 2021 American Chemical Society.

Recently, Li *et al.* developed three different BL platforms for the estimation and monitoring of O_2_˙^–^ radicals *in vivo*.^98^ The developed probes relied on attaching the OH moiety of d-luciferin with three different identification moieties for the selective recognition and detection of O_2_^•–^ (Figure 5A). They used *p*-nitrobenzenesulfonyl, trifluoromethanesulfonate, and diphenylphosphinyl as recognitions groups for constructions of probe F, probe N and probe P, respectively. According to *in vitro* studies, probe P was the best of the three probes for detecting O_2_^•–^ with high sensitivity, great selectivity, and excellent stability. During cisplatin treatment, probe P was effectively used for real-time observation of O_2_^•–^ in both cancer cells and tumors. The imaging results showed that the quantity of O_2_^•–^ in cancer cells rose with increasing cisplatin dosage.

Detecting and tracking ONOO^–^ in living cells is still challenging owing to the high autofluorescence background and the incomplete light penetration ability. Zhang’s group proposed a BL probe that relied on luciferase-luciferin pairs and used an ONOO^–^ recognition moiety (α-ketoamide) for the selective estimation and visualization of intracellular ONOO^–^ in living cells and mice.^99^ ONOO^–^ could react with the α-ketoamide moiety presented onto a luciferase substrate to produce aminoluciferin and then produce BL in the presence of luciferase. The probe has displayed high sensitivity for imaging the target sample in living cells and tumors. Furthermore, they employed the developed probe for visualizing the ONOO^–^ species in living mice model of inflammation.

### 4.4 BL Probes for the Detection of Proteins

Chen and co-workers designed a novel BL probe for the selective determination of urokinase-type plasminogen activator (uPA) activity for the first time.^100^ As shown in Figure 5B, they have blocked d-aminoluciferin by Gly-Gly-Arg to produce nonluminescent Gly-Gly-Arg-d-aminoluciferin (GGR-AmLuc). The developed probe, GGR-AmLuc, could selectively react with uPA to release d-aminoluciferin which gives high BL intensity in the occurrence of luciferase enzyme and other cofactors. It has been used for the selective sensing of uPA with a LOD of 1.37 μg/L. Finally, GGR-AmLuc was applied for tracking uPA activity in cancerous tissues of living mice. As seen in Figure 5B, the BL response of the investigated tumors improved noticeably, reached its plateau after 15 min, and then decreased, indicating a rapid reaction of GGR-AmLuc toward uPA to produce aminoluciferin for subsequent BL generation. While the uPA activity of the control group was inhibited, the BL signals intensity from the tumors reached its plateau after 5 min and then diminished rapidly. The attained findings demonstrated that the significant BL response in the investigational group was typically caused by uPA cleavage of the established GGR-AmLuc probe, and the probe could be effectively used to track uPA activity in tumors.

**Figure 6:**
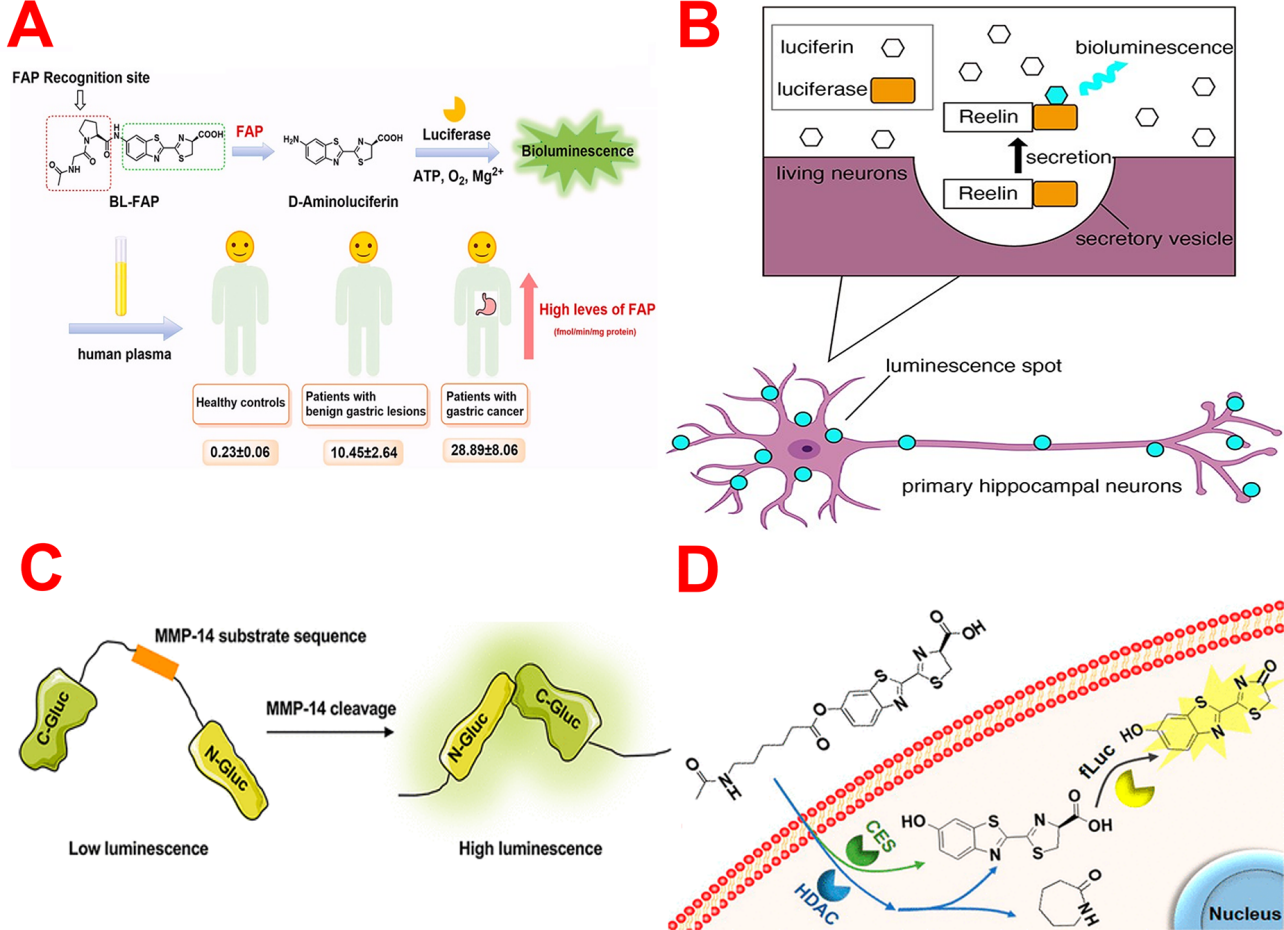
(A) Schematic illustration shows the design strategy of FAB BL probe. Reprinted with permission from ref (103). Copyright 2022 Elsevier. (B) Imaging of reelin by BL technique. Reprinted with permission from ref (105). Copyright 2022 Oxford University Press. (C) Diagram shows the MMP-14 biosensor. Reprinted from ref (106). Copyright 2021 American Chemical Society. (D) *In vivo* release of firefly luciferin for in vivo visualization of CES and HDAC activity. Reprinted from ref (111). Copyright 2020 American Chemical Society.

As a critical component of the cell surface glycoprotein family, FAP is abundant in reactive fibroblasts of malignancies and many fibrosis disorders. Lin *et al.* were the first to synthesize a BL platform for the estimation and *in vivo* imaging of FAB.^101^ They used *N*-carbobenzyloxy-Gly-Pro-OH (Cbz-Gly-Pro-OH, probe I) and *N*-*tert*-butoxycarbonyl-Gly-Pro-OH (Boc-Gly-Pro-OH, probe II) for blocking the aminoluciferin luminophore. The utilized groups were applied as the identification groups for FAP detection. In this study, probe I has shown a LOD of 0.254 ng/mL that could be utilized for the *in vitro* testing of FAP inhibitors. Also, Probe I was used for FAP estimations in living cells as well as in living mice. The developed method is anticipated to offer an exceptional chemical way to sensitively and selectively monitor the FAP levels and improve the authors’ perception of this protein’s biological relevance. Similarly, FAP was visualized by a novel amphiphilic block copolymer-based BL platform.^102^ The established platform was based on a micelle form (peptide-linked amphiphilic block, PABC) for long-standing *in vivo* monitoring of the FAP activity. The abundance of d-luciferin species was entrapped into a FAP micelle to create the BL platform. As a result of the barrier impact of block copolymers, the d-luciferin was isolated from luciferase to retain the PABC in the “signal off” mode. The activated polymer would pass through the cell and be destroyed by the intracellular FAP, which would break the micelle and release internal d-luciferin for subsequent enzymatic activity. It is difficult to pump out the prepared PABC by live cells due to its nanoscale size, and therefore the circulation time would be lengthened. Furthermore, because of its large loading capacity, the active micelle will act as a depot in this phase, resulting in a gradual and prolonged release of d-luciferin. In the existence of luciferase, ATP, O_2_, and Mg^2+^, unrestrained d-luciferin would be converted into oxyluciferin, allowing for long-term detection of FAP and *in vivo* observing of FAP. Moreover, Zhang and co-workers proposed a new BL probe for tracking FAB *in vivo* and in human plasma.^103^ The developed probe design strategy relied on attaching N-acetyl-Gly-Pro-OH (Ac-Gly-Pro-OH) as a FAP recognition group to the amino moiety of aminoluciferin (Figure 6A). This probe has revealed excellent selectivity as well as sensitivity (LOD= 18.1 pg/mL). The established FAP probe enables the endogenous tracking of FAP in living cells as well as nude mice bearing MGC-803-luc tumors. What’s more, this probe was effectively utilized for estimating the levels of the target analyte activity in plasma samples from patients suffering from gastric cancer as a first attempt. Notably, FAP levels were found to be significantly higher in patients with gastric cancer, proposing that FAP levels may be a possible pinpointing indicator for gastric cancer.

Yang and co-authors proposed different BL biosensors for the determination and *in vivo* imaging of nitroreductase (NTR) in hypoxic cancers.^104^ The probes’ strategy is based on the mechanism that the target analyte promotes the reduction of the nitrobenzyl group to the aniline moiety with an electron donor, thus resulting in 1,4 or 1,6-rearrangement–elimination to give coelenterazine analogs, which can be catalyzed by Renilla luciferase to generate BL response. Probes A1, A2, and A5 demonstrated good specificity in a dose-dependent way. Furthermore, probe A5 has exhibited excellent sensitivity, minimal cytotoxicity, and acceptable compatibility, allowing it to be effectively used to detect hypoxia state *in vitro* and *in vivo* as the first used coelenterazine-type BL approach.

Reelin is a glycoprotein that is essential for adult brain growth and synaptic plasticity. According to the literature, reelin is produced by nerve terminals and acts as a neurotransmitter. The reelin excretion mechanism remains uncertain. In their research, Nakao and co-workers could observe reelin release by BL imaging technique utilizing a fusion protein of reelin and gaussia luciferase (GLase-reelin).^105^ GLase-reelin expressed in HEK293T cells was properly processed and secreted. BL responses from the released GLase-reelin of primary cultured neurons were imaged by BL microscopy (Figure 6B). Notably, reelin secretory spots were detected at neuritis as well as cell bodies.

### 4.5 BL Probes for the Detection of Enzyme Activities

Matrix metalloproteinase-14 (MMP-14) regulates cancer migration and metastasis by directing extracellular matrix remodeling and cell motility. Despite intense attempts to create a method for detecting MMP-14 expression, there is a shortage of equipment able to estimate MMP-14 activity in living cells and animals with high temporal and spatial precision. Herein, Tian *et al.* constructed a novel BL probe for imaging MMP-14 activity based on the construction of Gaussia luciferase (Gluc)-based membrane-bound platform.^106^ As seen in Figure 6C, they added a platelet-derived growth factor receptor (PDGFR) transmembrane domain to the C-terminus of Gluc in order to anchor the MMP-14 reporter to the cell membrane. To create the MMP-14 membrane-bound reporter, the MMP-14 protease recognition sequence was inserted into the circularly permuted (CP) Gluc protein. Upon cleavage of MMP-14, high BL signal intensity was obtained and enabled imaging of MMP-14 activity in tumor-bearing mice with great selectivity and detection sensitivity. The above outcomes revealed that this biosensor is a suitable BL probe for detecting proteolytic activities in live cells. These outcomes will open the way for designing several probes based on using membrane-fixed proteases in living models. Won’s group used the commercial NpFlamma BL probe to visualize MMP-2 and MMP-9 activities in the corneal damage of mice induced by ethanol.^107^ It is well-known that MMP-2 and MMP-9 elevated activities are directly correlated with each step of ocular surface damage and can result in prolonged corneal injury healing. NpFlamma MMP-2,9 is a red dye-incorporated MMP-activatable chitosan nanoparticle (CNP) that enables the selective detection of MMP-2 and MMP-9 activities. The probe comprises Flamma dye that is linked to a quencher *via* an MMP-2,9 releasable peptide, and the peptide is chemically conjugated to CNP. These probes have been successively applied for monitoring and imaging of MMP-2,9 after ethanol-induced corneal injury and checking the efficiency and mechanism of drug interaction in the case of corneal epithelial syndromes.

To quantify γ-glutamyl transpeptidase (GGT) activity exogenously and endogenously, Lin and co-workers established a sensitive BL biosensor for achieving this purpose.^108^ They have selected aminoluciferin (which has a high BL wavelength at around 596 nm and excellent compatibility with deep tissue imaging) as the basic substrate for the next modifications. γ-Glutamyl moiety was used as a recognition group for attaching to aminoluciferin to form an amide bond. The as-prepared BL probe cannot be identified by the luciferase to generate the BL response. However, in the presence of GGT, the caged moiety could be cleaved selectively by the enzymatic hydrolysis reaction to give aminoluciferin that can generate intense BL through its subsequent reaction with firefly luciferase. It demonstrated a 309-fold increase when exposed to 100 U/L GGT and showed excellent selectivity toward inorganic and biological interfering compounds. In addition, it has been used for visualizing endogenous GGT activity in mice. When GGT activity was inhibited by 6-diazo-5-oxo-l-norleucine (DON), the BL intensity of the developed probe has been decreased, indicating its suitable application for *in vivo* imaging of the activity of GGT in nude mice.

Lin and colleagues developed a novel BL probe for the estimation and *in vivo* imaging of pantetheinase (a glycosylphosphatidylinositol enzyme) that is overexpressed in inflammation conditions and some metabolic diseases.^109^ The proposed probe was based on caging the amino group of luciferin with pantothenic acid. In this situation, pantetheinase can break the amide bond, releasing aminoluciferin, which is then recognized by firefly luciferase, resulting in the emission of a BL signal. The developed probe has been employed for the sensitive quantification of the target analyte with a LOD of 1.14 ng/mL. Moreover, animal trials verified that the proposed BL method can be employed to visualize the endogenous pantetheinase activity using pathogen-free luciferase-expressing transgenic mice. Notably, oral treatment of the pantetheinase-specific inhibitor (RR6) significantly suppressed BL intensity in transgenic mice. While starvation has increased the BL response of the transgenic mice due to an increase in pantetheinase activity. Furthermore, the proposed probe was used for the evaluation of pantetheinase activity in mouse tissue homogenates and plasma.

Cathepsin B (CB)-specific BL platform (Val-Cit-Luc) was established for the specific determination of CB activity with a 67-fold “signal-on” of BL response and a very low LOD of 27 mU/L.^110^ CB is a lysosomal protease that can cleave selectively Val-Cit/luciferin linker in the Val-Cit-Luc to produce aminoluciferin which is then catalyzed by luciferase in the occurrence of ATP, Mg^2+^ and oxygen to generate strong BL intensity. Inhibitory experiments showed that Val-Cit-Luc is an excellent method for monitoring CB activity in living cells and cancers. In the near future, Val-Cit-AL may be utilized for the diagnosis of CB-associated abnormalities in transgenic mice or for checking the CB functions in a boarder range of biological processes.

Recently, Wang and co-workers fabricated one BL probe for observing both the esterase (ES) and histone deacetylase (HAC) activity in living organisms.^111^ They synthesized acetamidohexanoic acid-d-luciferin (AcAH-Luc) for the concurrent visualization of ES and HAC activity with outstanding detection limits (LODs of 0.495 and 1.14 nM for ES and HAC6, respectively) and specificity (Figure 6D). AcAH-Luc was effectively used for the selective determination of ES and HAC6 (a subtype of HAC) offering linearity of 0–100 and 0–120 nM, respectively. *In vivo* studies showed that ES and HAC activity in the tumors generated around half and one-third of the “signal-on” BL response of AcAH-Luc, respectively.

Three BL probes (SBPs) for the first time have been synthesized by Yang and co-authors for *in vivo* imaging of sulfite oxidase deficiency.^112^ The recognition mechanism between SBPs and sulfite relied on the sulfite-mediated intramolecular cleavage interaction. Among them, SBP-1 probe displayed excellent reactivity, high selectivity and sensitivity. Based on these merits, the first *in vivo* visualization of sulfite levels in a living animal has been effectively attained.

Moreover, Luo *et al.* established a novel BL probe for the *in vivo* observing the hyaluronidase 1 (Hyal-1) activity and this probe could differentiate Hyal-1 from other isoforms.^113^ The proposed approach relied on the presentation of a noncovalently caging method to create a Hyal-1-selective BL platform by trapping and capturing d-luciferin into the cholesterylamine-modified hyaluronic acid (CHA) nanoassembly to hinder the BL generation. When CHA came into contact with intracellular Hyal-1, it decomposed completely, releasing several molecules of the entrapped d-luciferin and generating light emission *via* the luciferase enzyme-catalyzed BL process. The “signal-on”-type probe has been effectively used for the determination of Hyal-1 with high sensitivity (LOD= 0.07 ng/mL). Furthermore, BL imaging studies demonstrated that d-Luc@CHA was capable of imaging the Hyal-1 alterations in living cells and animals, as well as distinguishing between normal and malignant cells.

Yang *et al.* developed a novel BL biosensor for sensing the activity of two important analytes namely, alkaline phosphatase (ALP) and γ-glutamyltranspeptidase (GGT) *in vitro* and *in vivo*.^114^ The designed platform was constructed for real-time monitoring and specific cancer visualization through the cocleavage of the two analytes (ALP and GGT). They constructed the BL probe based on using d-luciferin which contains *p*-hydroxymethylphenyl and phosphate as self-cleavable moieties for the assay of ALP and GGT, respectively. The determination limits of the constructed probe were found to be 0.172 and 0.634 U/L for ALP and GGT, respectively. Finally, the synthesized probe was positively employed for the selective visualization of the co-overexpressed ALP and GGT in fLuc-231 breast cancer cells.

Tyrosinase (TYR), a crucial enzyme in the production of melanin dye, is commonly used as a diagnostic indicator for serious skin conditions such as vitiligo as well as melanoma cancer. Precise and accurate *in vivo* estimation of its activity is critical, but it remains difficult. Taking the merits of the BL imaging technique and the selective hydroxylation of the 3-hydroxybenzyloxy moiety by TYR, Li *et al.* developed TYR-LH2 BL probe by the substitution d-luciferin with 3-hydroxybenzyl moiety.^115^ The established biosensor has been used for the assay of the target enzyme with high detection sensitivity (0.11 U/mL) and selectivity. They used the developed probe for the *in vivo* imaging of TYR using 2 groups of mice. One group was injected with kojic acid before the probe injection for the negative control test. The obtained images revealed that the fabricated platform was capable of recognizing the dynamic TYR variations as well as distinguishing melanocytes malignant cells from other healthy cells, opening up new avenues for the diagnosis of many TYR-associated abnormalities.

### 4.6 Other BL Probes

Lu’s group developed BL probe for the detection and visualization of carbon monoxide (CO) in living species and mice relying on the Tsuji–Trost reaction of allyl-luciferin (AL) probe.^116^ The established probe was dependent on using Pd^0^ as a catalyst for Tsuji–Trost reaction, so AL selectively responded to the CO target to gived-luciferin and consequently produce a “signal-on”-type BL platform. They have synthesized the “signal-off” mode of AL and employed it as an innovative BL platform. First they injected AL and liposomes that involved PdCl_2_ intraperitoneally and intratumorally. After that they injected [Ru(CO)_3_Cl-(glycinate)] to generate exogenous CO. Consequently, CO reduces PdCl_2_ to Pd^0^, and the Tsuji–Trost reaction mediated by Pd^0^ results in the formation of d-luciferin, prompting the actual visualization of CO. The constructed system has exhibited high specificity in the occurrence of several biomolecules. This “signal-on”-type BL probe was sensitive and selective to both exogenous as well as endogenous CO. Recently the same group proposed a new approach for detecting and imaging CO inside the cancer cell.^117^ Herein, they modified d-luciferin with a heavy iodine atom to diminish its luminescence efficiency through the intersystem crossing process. This design strategy enables the establishment of a “signal-on” BL probe for CO monitoring. In this work, CO catalyzes the reduction of Pd^2+^ ions to Pd^0^ metal. Then, the generated Pd^0^ encouraged the specific deiodination reaction of 7′-iodo-luciferin to release free d-luciferin which subsequently reacts with luciferase to emit a BL signal. The proposed probe has displayed excellent selectivity and low cytotoxicity and good biocompatibility that allow the exogenous detection of CO produced by CORM-3 and visualization of the *in vivo* CO using the developed BL imaging technique.

An effective imaging-based monitoring approach that combines long persistent luminescence nanoparticles (LPLNPs) and red-emitting firefly luciferase (RfLuc) was developed to investigate the performance of the implanted human mesenchymal stem cells (hMSCs) which are significant in the pulmonary fibrosis therapy.^118^ LPLNP (Zn_1.1_Ga_1.8_Ge_0.1_O_4_:Cr^3+^, Eu^3+^) were reasonably fabricated, and the Cr^3+^ doping level could make the persistent luminescence (PL) of the LPLNP restorable with a tissue-penetrating red light rather than UV light and diminishing the impact of the PL decay lifetime. Also, the Cr^3+^-doped LPLNP was covered with a poly-l-lysine layer (LPLNP@PLL) *via* electrostatic attraction to increase the cellular uptake of the fabricated LPLNP for improving PL tracking of the target hMSCs. Furthermore, the endogenic RfLuc was used for the *in vivo* tracking of the living hMSCs. Finally, the movement, position, and survival of intravenously injected hMSCs in the PF mouse could be visualized *in vivo* by the simultaneous recording of the NIR-PL and BL responses.

Norepinephrine (NE) is a neurotransmitter that is essential for psychiatric illnesses, neurodegenerative abnormalities, fibromyalgia and pheochromocytoma. A noninvasive approach was developed for detecting NE based on using *p*-toluene thiol as a recognition group for the selective determination of NE by nucleophilic substitution reaction.^119^ In the *in vitro* study, the NE probe displayed reasonable sensitivity and specificity toward other interfering species including dopamine, GABA, and various amino acids, for instance, Ser, Lys, Cys, Thr, Hcy, and GSH. More significantly, the established probe was effectively used for imaging NE in transgenic mice, thus offering a prospective method for the noninvasive imaging and real-time monitoring of NE in living animals.

In comparison to d-luciferin and aminoluciferin, 2-hydroxyethyl luciferin (HE-Luc) may generate more effective and long persistent BL responses, making it a better choice for methods requiring longer integration periods, including 3D animal BL imaging. However, the complexity of the synthesis steps for HE-Luc suggests the few HE-Luc-based BL probes in the literature. Li and co-authors synthesized an effective HE-Luc BL probe through a facile method for monitoring intracellular hydrogen sulfide (H_2_S) in malignant cells for the first time.^120^ The synthesized probe has shown a high yield of 64% as compared with the 15% earlier synthesized compound. Fabricating HE-Luc as a small molecular probe (*R*)-2-(6-((2-((2-((2,4dinitrophenyl)thio)benzoyl)oxy)ethyl)amino)benzo[*d*]thiazol-2-yl)-4,5-dihydrothiazole-4-carboxylic acid (DNPT-HS) was intended for the estimation of H_2_S both *in vitro* as well as *in vivo*. Notably, DNPT-HS had outstanding detection ability in the luciferase-transfected malignant cells and nude mice, and the S/N ratios of DNPT-HS were estimated to be 26, 21, and 7 for Huh7 cells, MDA-MB-231 cells, and mice, respectively, which was the most effective BL platform for visualizing H_2_S *in vivo*. Also, the developed probe has displayed excellent selectivity toward several potential interference species. Also, for recognizing hydrogen polysulfides in a murine model of bacterial infection and live systems, Li *et al.* developed a selective BL probe for this purpose.^121^ They created the probe by incorporating an H_2_S_*n*_ recognition group into a d-luciferin molecule. The probe can be transformed to d-luciferin through a selective response with H_2_S_*n*_, producing high BL intensity. The excellent biocompatibility of the proposed probe enables its application for imaging the fluctuations in H_2_S_*n*_ level in living cells. Furthermore, it was employed for imaging of the endogenous H_2_S_*n*_ in bacterially infected living mice.

By comparing the reported BL probes in this review, we noticed that most of the reported BL probes were based on using d-luciferin and amino-luciferin units rather than coelenterazine-based BL probes for developing a “signal-on”-type BL probes for imaging of various analytes. We think the reason for this is that coelenterazine has poor aqueous solubility and the molecule is larger in size and has a higher toxicity than luciferin.^122^ Also, it is susceptible to auto-oxidation resulting in background CL in solution. Moreover, coelenterazine has a different transportation mechanism to the cell than luciferin. Furthermore, in comparison to d-luciferin and aminoluciferin with 2-hydroxyethyl luciferin (HE-Luc), HE-Luc may generate more effective and persistent BL responses, making it a better choice for methods requiring longer integration periods, including 3D animal BL imaging. However, the complexity of the synthesis steps for HE-Luc suggests the few HE-Luc-based BL probes in the literature. Therefore, simple synthetic procedures for HE-Luc should be developed in the future.

## 5 Conclusion and Future Perspectives

CL and BL detection approaches are characterized by their excellent signal-to-noise ratio (S/N), high sensitivity, wide linearity, fine rapidity and are techniques of choice in real-time noninvasive imaging. Therefore, BL and CL have been widely employed as detection approaches in many fields, including immunology, genetics, pharmaceutical analysis, cytology, forensic medicine, liquid chromatography, and imaging technology. CL and BL imaging approaches have substantially evolved with the development of novel luminous substances and systems, as well as their integration with other methods like ELISA and molecularly imprinted polymers. Moreover, BL and CL imaging microscopy exhibit the merits of high detectability, selectivity, and easy estimation of the luminescent signal response for carrying out a simple and accurate quantitative assay of the labeled probes, which is considered to be a significant advantage over the traditional fluorescence-based imaging technology.

Despite the huge progress and implementations of CL and BL, many issues remain to be addressed in future research. For example, most of the CL probes are based on using 1,2-dioxetane derivatives as activatable platforms for various substrates; however, their relatively complex fabrication routes have hampered their wide utilization. Therefore, it is essential to propose simple methods for their synthesis as activatable CL probes for imaging applications. Also, compared to the visible region, NIR (700–900 nm) and the second near-infrared (NIR-II) regions (1000–1700 nm) display inherent merits, for instance, deeper tissue penetration and a higher SNR. So, it is encouraging to establish modifiable CL probes in the NIR area for future CL-based bioimaging. Moreover, the utilized CL scaffolds still have short half-lives; accordingly, the fabrication of new scaffolds with great quantum yields (QY) and persistent emission may be useful to extend the CL applications, especially in PDT therapy.^123−125^

On the other hand, caged luciferins have been extensively employed as BL platforms for several applications, for example, the detection of diseases-related bioactive markers, metal ions detection, and pharmaceutical analysis. However, the emission spectra of most of the fabricated caged luciferins appear in the visible range and therefore overlap with the absorption spectra of certain naturally occurring *in vivo* biomolecules, hence deep tissue imaging based on these fabricated caged luciferins becomes difficult and challenging.^126^ As a result, the establishment of d-luciferin derivatives with long emission wavelengths will greatly broaden the uses of BL probes in the imaging of deep tissues.^127^ Furthermore, in the event of future endeavors toward the synthesis of luminescent probes, special consideration should be paid to the fabrication of innovative and effective luciferin skeletons to spread out the applications of BL to a large extent since a large number of the derivatives can only be attained by structural modification of d-luciferin skeleton. Lastly, the progress in the fabrication of derived luciferase–luciferin pairs with bathochromic shift of emission spectra will enhance the penetration of BL through tissues and increase its uses in deep tissues imaging by reducing the optical signal attenuation.^36,81^

In the end, we think the research community will search for and develop new CL and BL-based imaging methods such as thermochemiluminescence (TCL) approaches to overcome the stability limitations of the CL and BL methods of imaging.^128−130^

## Vocabulary

complementary metal-oxide semiconductor (CMOS): a type of commonly used active-pixel image sensor, where each pixel sensor unit cell has a photodetector (typically a photodiode) and an amplification circuit built with complementary metal-oxide-semiconductor field-effect transistors
photodynamic therapy (PDT): treatment that involves light-sensitive medicine activated by a light source to destroy abnormal cells
pantetheinase: an enzyme responsible for the hydrolyses of d-pantetheine into cysteamine and pantothenate (vitamin B5) on the dissimilative pathway of CoA
thermochemiluminescence (TCL): a potentially simple and sensitive detection principle, as the light emission is simply elicited by a thermally triggered decomposition of a molecule to produce a singlet excited state product
L012: a luminol derivative and is widely used in physiological conditions to detect NADPH oxidase (Nox)-derived superoxide radicals and identify Nox inhibitors
